# KLRD1 (CD94): A Prognostic Biomarker and Therapeutic Candidate in Head and Neck Squamous Cell Carcinoma

**DOI:** 10.7150/jca.104762

**Published:** 2025-01-01

**Authors:** Chengyuan Dong, Ziyou Lin, Yunzhao Hu, Qun Lu

**Affiliations:** 1 Shanghai TCM-Integrated Hospital, Shanghai university of TCM, Shanghai, China; 2 Tongji University School of Medicine, Shanghai, China

**Keywords:** KLRD1, HNSC, Immune checkpoints, Independent prognostic factor

## Abstract

**Background:** Killer Cell Lectin Like Receptor D1 (KLRD1) plays a crucial role in antitumor immunity. However, its expression patterns across various cancers, its relationship with patient prognosis, and its potential as an immunotherapy target remain inadequately understood.

**Methods:** We analyzed KLRD1 expression across various cancer types using multi-omics data from The Cancer Genome Atlas (TCGA), Genotype-Tissue Expression (GTEx), and Gene Expression Omnibus (GEO) databases, correlating it with patient prognosis. Single-cell RNA sequencing data were employed to further explore KLRD1 expression in natural killer (NK) cells and exhausted CD8+ T cells (CD8Tex). Functional enrichment analyses using Gene Ontology (GO) and Kyoto Encyclopedia of Genes and Genomes (KEGG) identified the biological processes and pathways associated with KLRD1. Immune infiltration analysis, conducted via CIBERSORT, assessed the relationship between KLRD1 expression and immune cell infiltration within the tumor microenvironment. Furthermore, the Tracking Tumor Immunophenotype (TIP) meta-server and Easier tool were employed to assess the role of KLRD1 in the cancer immunity cycle and to predict immunotherapy responses. Drug sensitivity was predicted using tools like CellMiner and the Genomics of Drug Sensitivity in Cancer (GDSC) database to explore the link between KLRD1 expression and responsiveness to various anticancer drugs.

**Results:** KLRD1 exhibits significant differential expression and strong prognostic value across cancers, particularly as an independent prognostic factor in head and neck squamous cell carcinoma (HNSC). Single-cell analysis revealed high expression of KLRD1 in NK and CD8Tex cells, indicating its critical role in antitumor immune responses. Functional enrichment analyses showed that KLRD1 is involved in several immune-related signaling pathways, including NK cell-mediated cytotoxicity and T cell receptor pathways. Immune infiltration analysis further confirmed a positive correlation between KLRD1 expression and the infiltration of various immune cells. Moreover, higher KLRD1 expression in HNSC is associated with enhanced immune pathway activity, increased sensitivity to cell division inhibitors, and the identification of arachidonyltrifluoromethane as a potential compound to counteract its oncogenic effects.

**Conclusion:** In HNSC, KLRD1 is a key prognostic marker and potential target for personalized immunotherapy.

## 1. Introduction

Cancer remains one of the most significant global health challenges, with 19.3 million new cases and nearly 10 million deaths recorded in 2020 alone 1, 2. Tumor recurrence and metastasis remain the leading causes of death in cancer patients, with the reshaping of the immune microenvironment by tumor cells playing a crucial role in these processes, as well as in drug resistance 3. The introduction of immune checkpoint inhibitors (ICIs) in recent years has transformed cancer treatment by activating the immune system and enhancing anti-tumor responses, resulting in considerable extensions in patient survival 4-6. However, the clinical application of ICIs faces challenges such as low response rates and short durations of effective response 7. Therefore, identifying novel immune checkpoints and understanding their regulatory mechanisms has become a crucial strategy to overcome these obstacles.

KLRD1, also known as CD94, plays a critical role in immune surveillance by forming a receptor complex with NKG2 molecules 8-10. Many factors influence the expression of KLRD1 molecules, including cytokines such as IL-12 and IFN-gamma, viral infections, and tumor factors. Furthermore, KLRD1/NKG2A expression has been linked to autoimmune illnesses, infectious diseases, and a variety of cancers 11-15. Lentz *et al.* found that the KLRD1 molecule, an important pathway of the immune checkpoint HLA-E, could be used as a new therapeutic tool for immunotherapy of tumors 16, 17. Platelet-derived RGS18 shields circulating tumor cells from NK-mediated immune surveillance by binding to the immune checkpoint HLA-E:KLRD1-NKG2A, according to Liu *et al.* By blocking inhibitory signaling, immune clearance of CTCs inhibits tumor metastasis *in vivo* 18. Meanwhile, the KLRD1-NKG2 complex can clear tumor cells by increasing NK cell activation and killing, as well as preventing T-cell attack on their own tissues by suppressing T-cell immunity, so several drugs targeting the KLRD1-NKG2 complex are in research or clinical trials 19. Research into KLRD1 is essential for developing novel cancer therapies by improving immune system detection.

This study seeks to assess the potential of KLRD1 as a therapeutic target in tumor immunotherapy through an extensive pan-cancer analysis. We will examine KLRD1 expression patterns, single-cell landscape, and prognostic significance within TME. Additionally, we will explore its impact on immune landscape, resistance to immune therapies, and drug sensitivity. This comprehensive analysis aims to elucidate how KLRD1 modulates critical aspects of tumor biology and evaluate its potential for advancing future cancer treatments.

## 2. Materials and Methods

### 2.1 Data Sources and Preprocessing

This study utilized data from multiple publicly available databases, including TCGA, GEO, and GTEx database. RNA sequencing (RNA-Seq) data encompassing various cancer types were downloaded from the TCGA database, while normal tissue RNA-Seq data were obtained from the GTEx database to evaluate the differential expression. Standardization of the data was performed using log2(FPKM + 1) or log2(TPM + 1) transformations. To ensure data quality, low-expression samples and those with incomplete clinical information were excluded, followed by data normalization.

### 2.2 Differential Expression Analysis

The differential expression of KLRD1 across various cancer types was analyzed using the "limma" R package. To validate the reliability of the results, independent datasets were selected from the GEO database for verification.

### 2.3 Prognostic Analysis

The prognostic value of KLRD1 in different cancer patients was assessed using univariate and multivariate Cox proportional hazards regression models to calculate hazard ratios (HR) and their 95% confidence intervals (CI). Kaplan-Meier (KM) survival analysis was employed to compare overall survival rates between high and low expression groups.

### 2.4 Single-Cell RNA Sequencing Analysis

Multiple single-cell RNA-Seq datasets were analyzed, including LIHC_GSE140228, HNSC_GSE139324, and NSCLC_GSE127465. Raw data were filtered, standardized, and normalized, followed by the selection of highly variable genes and linear dimensionality reduction. Clustering analysis was then performed to identify cellular subpopulations.

### 2.5 Functional Annotation and Pathway Analysis

The "clusterProfiler" R package was used for GO and KEGG enrichment analyses. Gene Set Enrichment Analysis (GSEA) was utilized to assess KLRD1-related signaling pathways.

### 2.6 Tumor Immune Infiltration Analysis

Using tools such as CIBERSORT, TIMER, and ESTIMATE, the relative abundance of immune cells in the tumor microenvironment (TME) can be calculated to gain a better understanding of the immune landscape in cancers. Additionally, the potential involvement of KLRD1 in different stages of the antitumor immune response was further investigated using the Tracking Tumor Immunophenotype (TIP) tool.

### 2.7 Immune Checkpoint Correlation Analysis

Expression data for KLRD1 and immune checkpoint molecules were collected and visualized using heatmaps generated by the "ComplexHeatmap" R package. Spearman correlation coefficients were calculated to assess the relationships between KLRD1 and various immune checkpoint molecules.

### 2.8 Drug Sensitivity Analysis

To investigate the impact of KLRD1 expression on cancer drug sensitivity, a systematic analysis was performed using data from the GDSC and connectivity map (cMAP). Statistical analysis was conducted using the "pRRophetic" R package, which combined p-values and effect sizes to evaluate the association between KLRD1 expression and drug sensitivity. Additionally, stratified analyses were performed for different drugs to confirm the sensitivity differences associated with KLRD1 in specific treatments.

### 2.9 Statistical Analysis

All statistical analyses were conducted using R software (version 4.2.0). Continuous variables were compared using either t-tests for normally distributed data. Categorical variables were evaluated using chi-square tests, depending on sample size and distribution. For all analyses, a p-value of less than 0.05 was considered statistically significant.

## 3. Results

### 3.1 Validation of KLRD1 Expression and Its Genomic Implications

**Figure 1:**
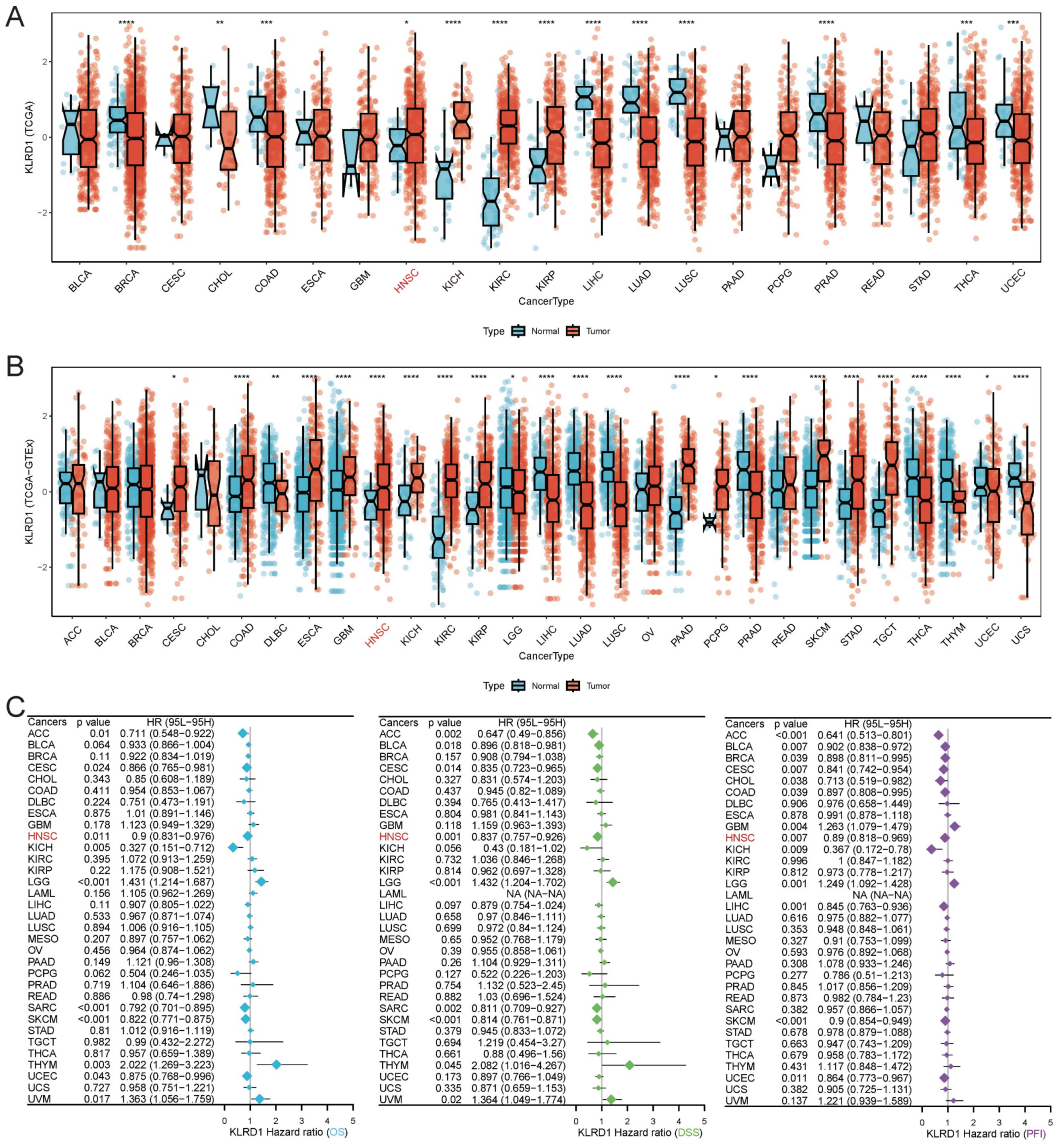
Differential Expression and Prognostic Value of KLRD1 Across Various Cancers. (A) Differential mRNA expression of KLRD1 across multiple cancers based on data from the TCGA database. Significance levels: *P<0.05, **P<0.01, ***P<0.001, ****P<0.0001. (B) Comparison of KLRD1 expression between tumor and normal tissues, combining data from TCGA and GTEx. Significance levels: *P<0.05, **P<0.01, ****P<0.0001. (C) Prognostic value of KLRD1 in different cancers, analyzed for Overall Survival (OS), Disease-Specific Survival (DSS), and Progression-Free Interval (PFI).

We analyzed KLRD1 gene expression using RNA sequencing data from TCGA, revealing significantly lower expression in breast invasive carcinoma (BRCA), cholangiocarcinoma (CHOL), colon adenocarcinoma (COAD), liver hepatocellular carcinoma (LIHC), lung adenocarcinoma (LUAD), lung squamous cell carcinoma (LUSC), prostate adenocarcinoma (PRAD), thyroid carcinoma (THCA), and uterine corpus endometrial carcinoma (UCEC), compared to normal tissues. Conversely, higher KLRD1 expression was noted in HNSC, kidney chromophobe (KICH), kidney renal clear cell carcinoma (KIRC) **(Figure 1A)**. Integrated analysis with GTEx data confirmed these patterns **(Figure 1B)**, emphasizing the potential clinical significance of KLRD1 as a biomarker. Univariate Cox survival analysis showed significant prognostic value for KLRD1 in several cancers, acting as a protective factor in adrenocortical carcinoma (ACC), cervical squamous cell carcinoma and endocervical adenocarcinoma (CESC), HNSC, low-grade glioma (LGG), and skin cutaneous melanoma (SKCM) **(Figure 1C)**. The validation using the GEO dataset (E_MTAB_8588) confirmed elevated KLRD1 expression specifically in HNSC **(Supplementary Figures 1A)**. Furthermore, within HNSC, higher KLRD1 expression correlated negatively with aneuploidy and ploidy scores **(Supplementary Figures 1B-D)**. In summary, these findings underscore the potential of KLRD1 as a valuable biomarker for HNSC and its association with genomic stability.

### 3.2 KLRD1 Expression in Single-Cell Landscapes

**Figure 2:**
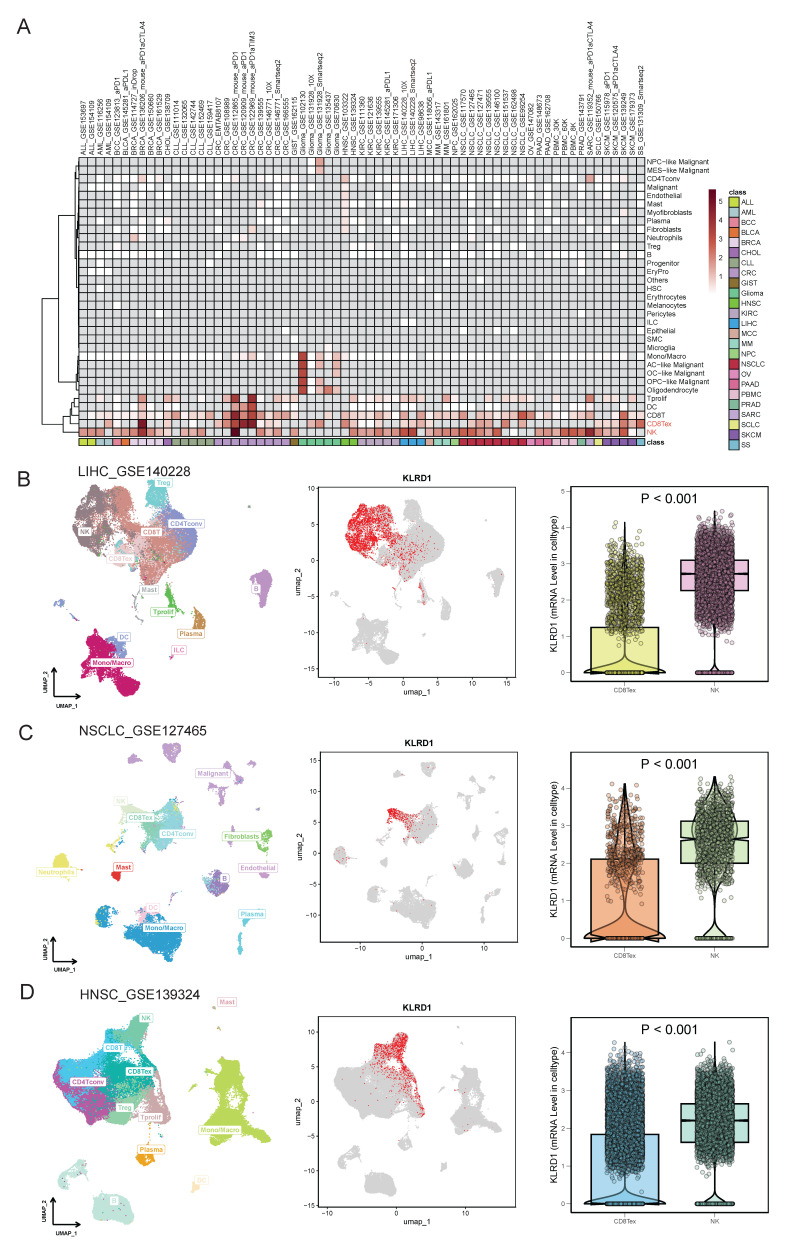
Single-Cell Expression Landscape. (A) Single-cell level expression of KLRD1 across various tumors. (B-D) Single-cell level expression of KLRD1 in liver cancer, small cell lung cancer, and head and neck cancer datasets. (Left panel) Immune cell expression profile across these tumors; (Middle panel) Distribution of KLRD1 expression in immune cells within these tumors; (Right panel) Comparison of the proportion of KLRD1 expression in CD8+ exhausted T cells (CD8Tex) and NK cells across these tumors.

Single-cell analysis revealed that KLRD1 is predominantly expressed in NK cells and CD8Tex cells across various cancers, particularly in bladder cancer (BLCA), HNSC, KIRC, non-small cell lung cancer (NSCLC), pancreatic cancer (PAAD), peripheral blood mononuclear cells (PBMC), and SKCM **(Figure 2A)**. Further validation through detailed analyses of datasets (LIHC_GSE140228, HNSC_GSE139324, NSCLC_GSE127465) demonstrated that KLRD1 predominantly clusters in NK cells, with significantly higher expression compared to CD8Tex cells **(Figures 2B-D)**. This consistent overexpression in NK cells highlights the role of KLRD1 as a marker for immune activity and a potential target in cancer immunotherapy.

### 3.3 Independent Prognostic Value of KLRD1 in HNSC

**Figure 3:**
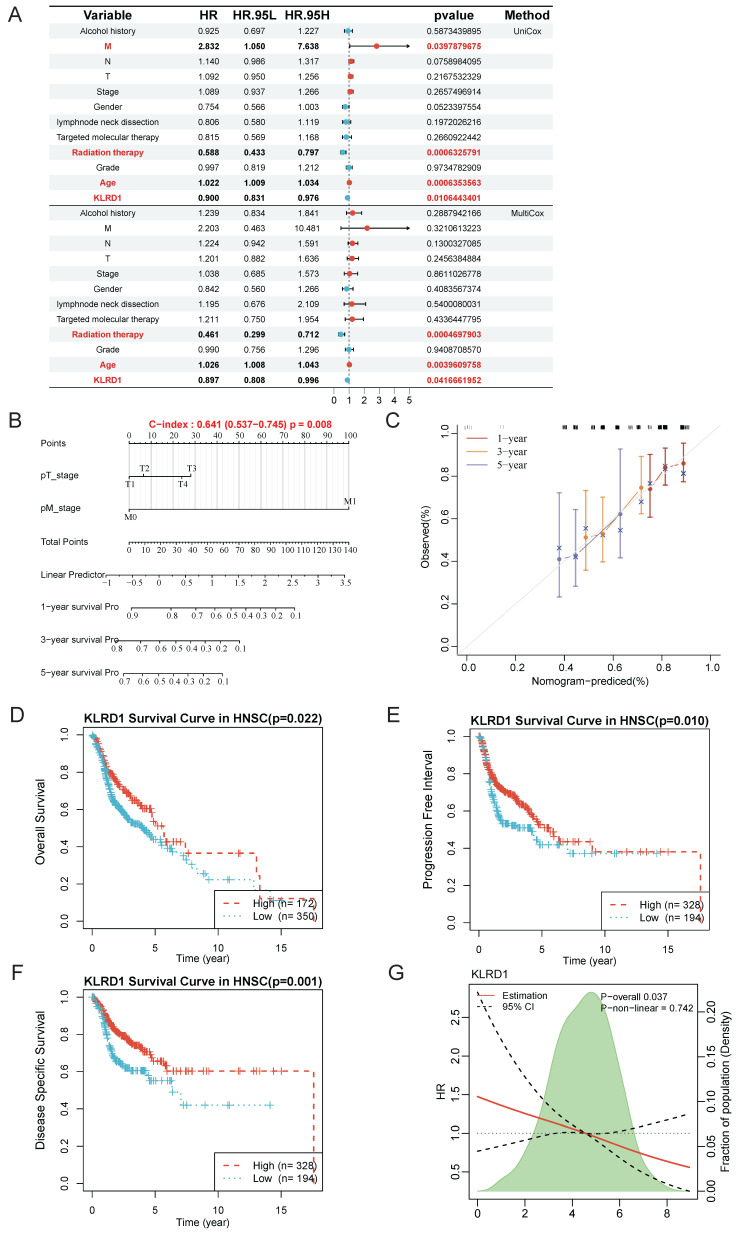
KLRD1 as an independent prognostic factor for HNSC. (A) Univariate Cox survival analysis is shown above the line, and multivariate Cox survival analysis is shown below. The relative risk is described using the hazard ratio (HR) and 95% confidence interval (CI). An HR greater than 1 indicates a risk factor, while an HR less than 1 suggests a protective factor. A p-value < 0.05 is considered statistically significant. (B) Nomogram construction. (C) Calibration plot of the nomogram. The calibration curves show the predicted 1-year, 3-year, and 5-year overall survival (OS) probabilities in the TCGA HNSC cohort. (D-F) Prognostic value analysis of KLRD1 for OS (D), DSS (E), and PFI (F). (G) Restricted cubic spline analysis to explore whether the risk associated with KLRD1 is non-linear across four survival outcomes (OS, DSS, PFI, DFI).

Given the significant differences in KLRD1 across differential analysis, genomic stability, and univariate prognostics, along with its predictive capability in single-cell analyses, we further explored its independent prognostic value and underlying molecular mechanisms. Univariate analysis revealed that M stage, radiation therapy, and age were significantly associated with the survival of HNSC patients, with KLRD1 emerging as a potential independent predictor of survival. This finding was corroborated by multivariate Cox survival analysis, which confirmed that radiation therapy and age were closely related to survival, while KLRD1 remained an independent predictor, regardless of other clinical factors (**Figure 3A**).

To further support our findings, we constructed a nomogram based on KLRD1 expression to enhance prognostic evaluation in clinical practice. Calibration curves showed that the predicted survival closely aligned with the ideal curve, indicating robust predictive performance (**Figure 3B-C**). Additionally, Kaplan-Meier survival analysis using data from the TCGA database further validated the prognostic capability of KLRD1, confirming that low KLRD1 expression is associated with poorer outcomes in HNSC patients (**Figure 3D-F**). The consistent linear relationship between KLRD1 expression and survival risk indicates that KLRD1 is a stable and reliable prognostic factor, making it a valuable biomarker for evaluating patient prognosis across diverse patient groups (**Figure 3G**). These results suggest that KLRD1 may play a crucial role in the pathophysiology of HNSC.

### 3.4 Pathway-Level Analysis and Functional Insights into KLRD1 in HNSC

**Figure 4:**
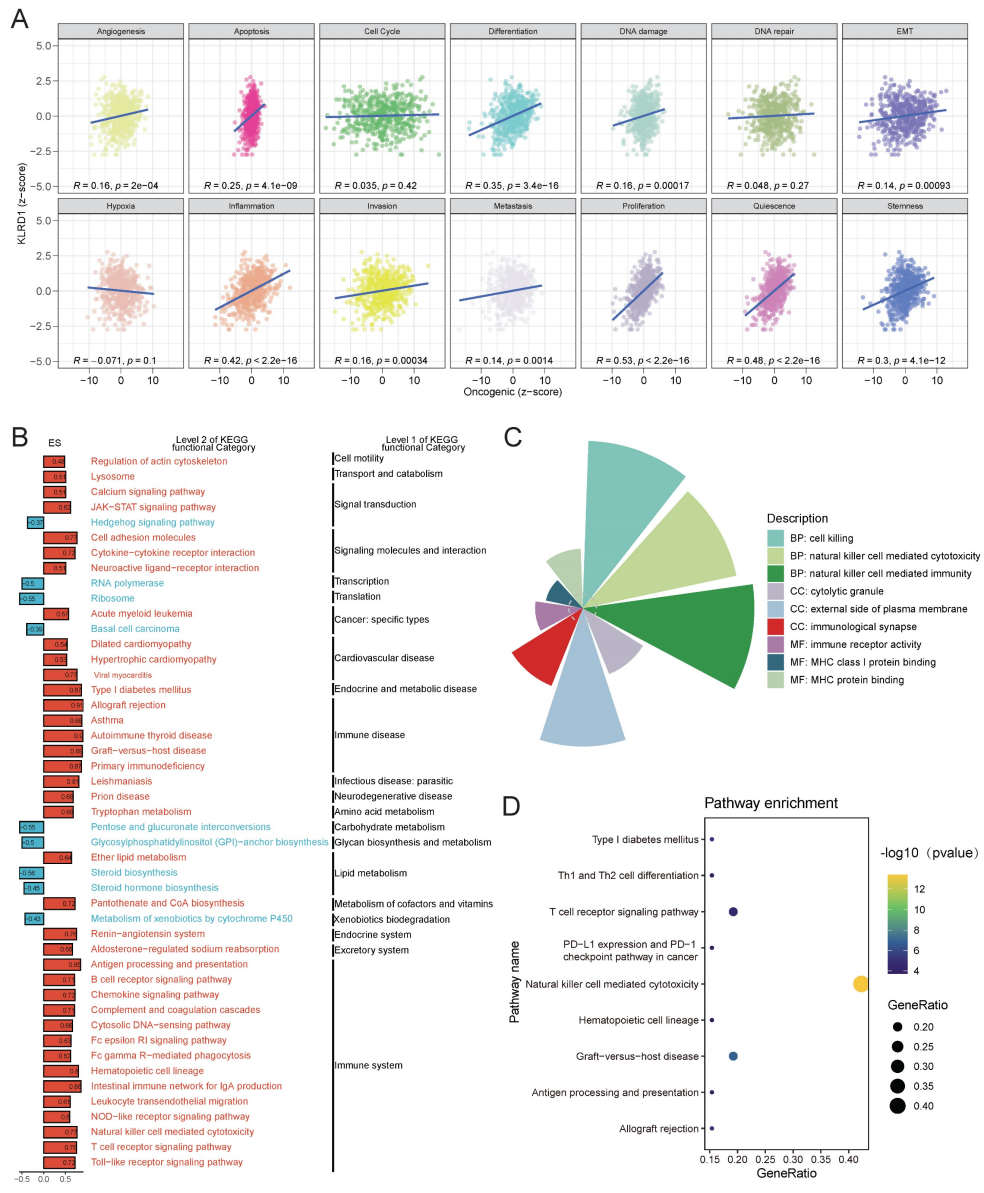
(A) Correlation analysis between KLRD1 expression and various oncogenic pathways, including angiogenesis, apoptosis, cell cycle, DNA repair, and more, across different cancers. (B) KEGG pathway enrichment analysis of KLRD1, showing significant associations with immune-related and signaling pathways. Pathways are categorized at Level 2 and Level 1 of KEGG functional hierarchy. (C) Functional categorization of biological processes influenced by KLRD1, particularly focusing on immune response mechanisms. (D) Pathway enrichment analysis highlighting key immune-related pathways associated with KLRD1, such as PD-L1 expression, T cell receptor signaling, and natural killer cell-mediated cytotoxicity. The GeneRatio and -log10(p-value) are used to assess pathway significance.

Transitioning from gene-level analysis to pathway-level analysis allows for more biologically meaningful insights and provides greater interpretability of life phenomena. By analyzing the Pearson correlation between gene expression z-scores and GSVA scores for 14 tumor-related states, it was found that in HNSC, KLRD1 is positively correlated with the activity of several pathways, including angiogenesis, apoptosis, differentiation, DNA damage, epithelial-mesenchymal transition, stemness, quiescence, proliferation, metastasis, invasion, and inflammation (**Figure 4A**). To further explore these relationships, samples were divided into high and low expression groups based on KLRD1 expression levels, with the top 30% defined as the high-expression group and the bottom 30% as the low-expression group. GSEA was then performed using the KEGG gene sets. The analysis revealed that pathways related to the immune system and immune-related diseases were significantly enriched in the KLRD1 high-expression group. These pathways include the renin-angiotensin system, immune checkpoint signaling, NK cell-mediated cytotoxicity, asthma, and allograft rejection. Additionally, pathways associated with the excretory system, endocrine and metabolic diseases, cardiovascular diseases, and signaling molecules and interactions were significantly enriched in the KLRD1 high-expression group (**Figure 4B**).

KLRD1 high expression is typically associated with synchronous changes in multiple genes, impacting various aspects of cellular signaling, gene expression regulation, cell differentiation, and disease progression. To gain a deeper understanding of the functional roles of KLRD1, we conducted GO and KEGG functional enrichment analyses of its co-expressed genes. The GO analysis revealed significant enrichment in cell killing, NK cell-mediated cytotoxicity and immunity, cytolytic granules, external side of the plasma membrane, immunological synapse, immune receptor activity, MHC class I/protein binding. These findings suggest that KLRD1 is involved in immune-related processes, particularly those linked to NK cell functions and immune synapse formation. KEGG pathway analysis further supported these findings, showing significant enrichment in pathways such as NK cell-mediated cytotoxicity, T cell receptor signaling pathway, and graft-versus-host disease (**Figure 4C-D**). Thses insight broadens our understanding of the impact KLRD1 has on cancer biology.

### 3.5 The Contribution of KLRD1 to Immune Infiltration in HNSC

**Figure 5:**
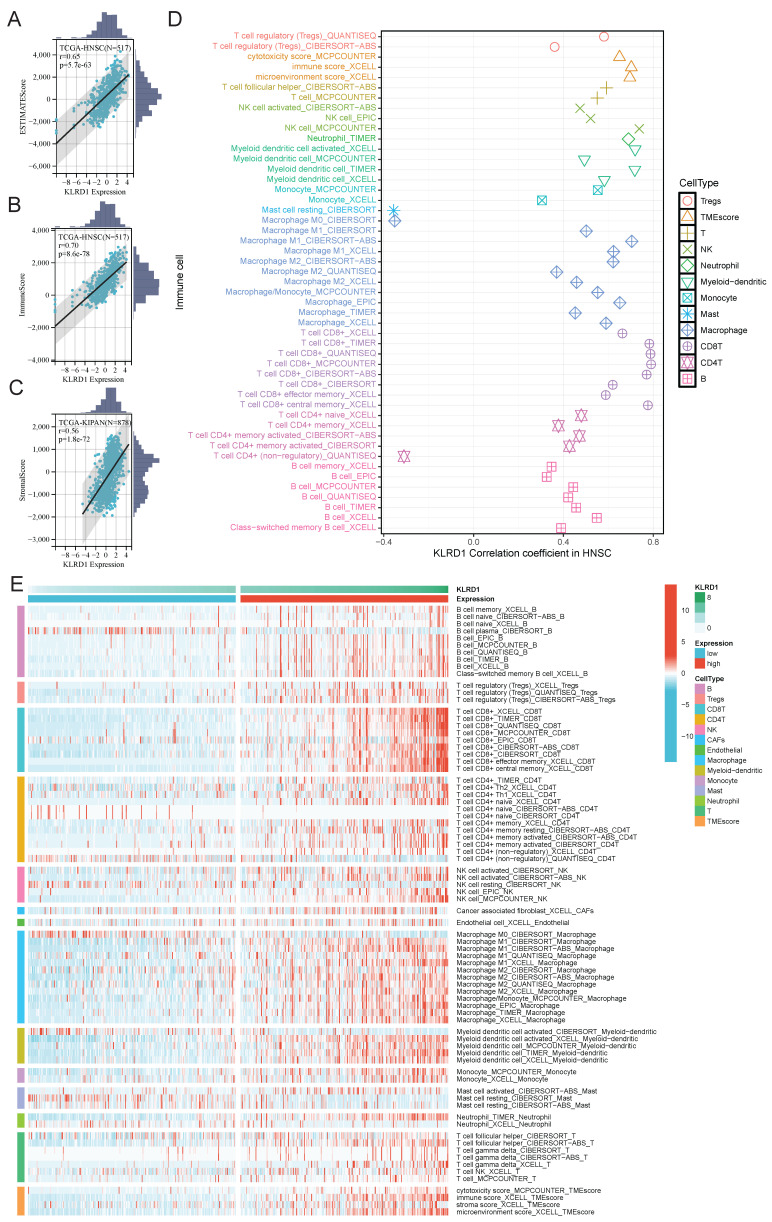
Correlation Analysis of KLRD1 with Immune Cell Infiltration in HNSC. (A-C) Correlation between KLRD1 expression and ESTIMATE score (A), ImmuneScore (B), and StromalScore (C) in TCGA-HNSC samples. (D) Correlation analysis between KLRD1 expression and infiltration levels of various immune cell types. (E) Heatmap showing the infiltration levels of different immune cells in HNSC across high and low KLRD1 expression groups.

Building on pathway-level insights into KLRD1, we investigated its role in immune infiltration within the HNSC microenvironment. We analyzed the association of KLRD1 with estimated scores, immune scores, and stromal scores in HNSC (**Figure 5A-C**). The results showed significant positive correlations, with correlation coefficients of 0.65, 0.70, and 0.56, respectively. Additionally, further analysis revealed a strong association between KLRD1 and various immune cells, including T cells, B cells, NK cells, myeloid dendritic cells, CD8+ T cells, M1 macrophages, and M2 macrophages. To explore these immune mechanisms in greater detail, we divided HNSC patients into high and low KLRD1 expression groups. In the high-expression group, KLRD1 remained significantly correlated with the infiltration of these immune cells, further supporting its role in immune infiltration (**Figure 5D-E**). These findings suggest that KLRD1 may provide new opportunities for developing targeted immunotherapies in HNSC.

### 3.6 KLRD1 in the Immune Landscape and Subtype Characteristics of HNSC

**Figure 6:**
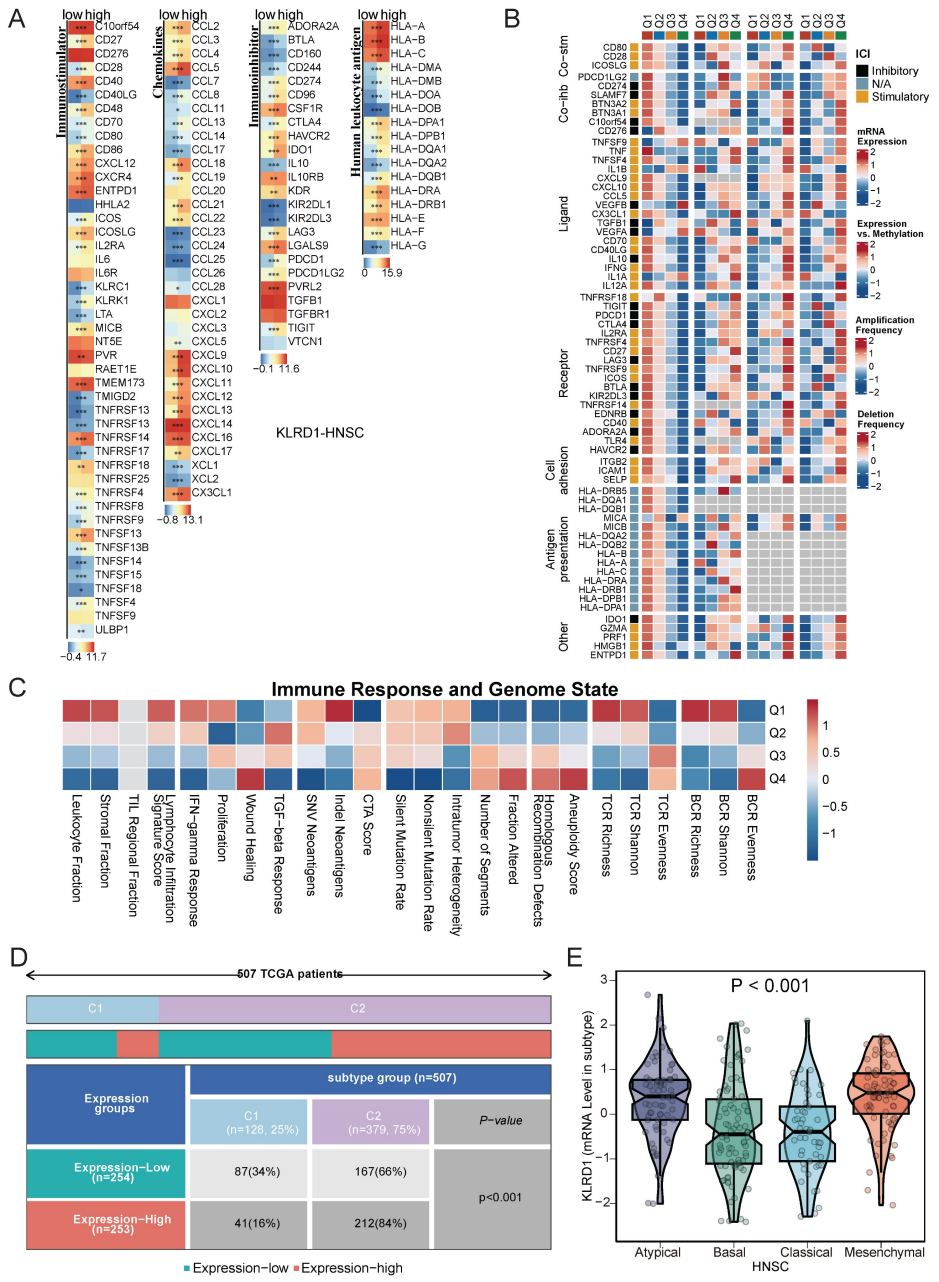
Analysis of KLRD1 in Immune Response and Genomic State in HNSC. (A) Heatmap displaying the differential expression of immune checkpoint molecules, chemokines, and HLA genes at varying levels of KLRD1 expression in HNSC. (B) Correlation analysis between KLRD1 expression and the expression, methylation, amplification, and deletion frequencies of immune checkpoint inhibitors and stimulators in HNSC. (C) Heatmap of the correlation between KLRD1 expression and immune response as well as genomic state in HNSC. (D) Correlation analysis between KLRD1 expression and HNSC subtype classification based on TCGA data. (E) Differences in KLRD1 mRNA expression levels among various HNSC subtypes.

To further explore the role of KLRD1 in the immune landscape of HNSC, we analyzed its association with immune checkpoints and subtypes, aiming to understand its link to key immune checkpoint regulation involved in immune evasion and tumor progression. Significant differences between high and low KLRD1 expression groups were observed in immune-stimulatory genes, immune-inhibitory genes, chemokines, and HLA molecules. In the high KLRD1 expression group, genes like C10orf54, TMEM173, CXCL9-17, HLA-A-C, and PVRL2 were upregulated, along with CD40LG, TMIGD2, TNFRSF13B, TNFSF14, CCL23-24, CCL7, KIR2DL1, and KIR2DL3 (**Figure 6A**). High KLRD1 expression was associated with elevated immune regulatory molecule expression, somatic copy number alterations (SCNAs), and epigenetic changes, particularly in the HLA family (**Figure 6B**). This high expression corresponds to an intricate immune regulatory landscape, including increased leukocyte and stromal fractions, lymphocyte infiltration, TCR and BCR diversity, and a strong interferon-gamma response. Conversely, low KLRD1 expression correlated with higher genomic instability, active wound healing processes, and a reduction in immune response diversity (**Figure 6C**).

Immune subtype analysis revealed that high KLRD1 expression is predominantly found in the C1 and C2 subtypes, with C1 linked to tissue healing and C2 dominated by interferon-gamma signaling, crucial for antitumor immune responses. KLRD1 expression was also notably elevated in Atypical and Mesenchymal subtypes, which are associated with unique molecular features, increased invasiveness, and therapy resistance. In contrast, lower KLRD1 expression was observed in the Basal and Classical subtypes, which have different molecular and clinical characteristics (**Figure 6D-E**). This distribution suggests that KLRD1 plays varied roles depending on the tumor's immune context and molecular profile, influencing both tumor progression and its interaction with the immune system.

### 3.7 Anticancer Immune Response and Drug Sensitivity

**Figure 7:**
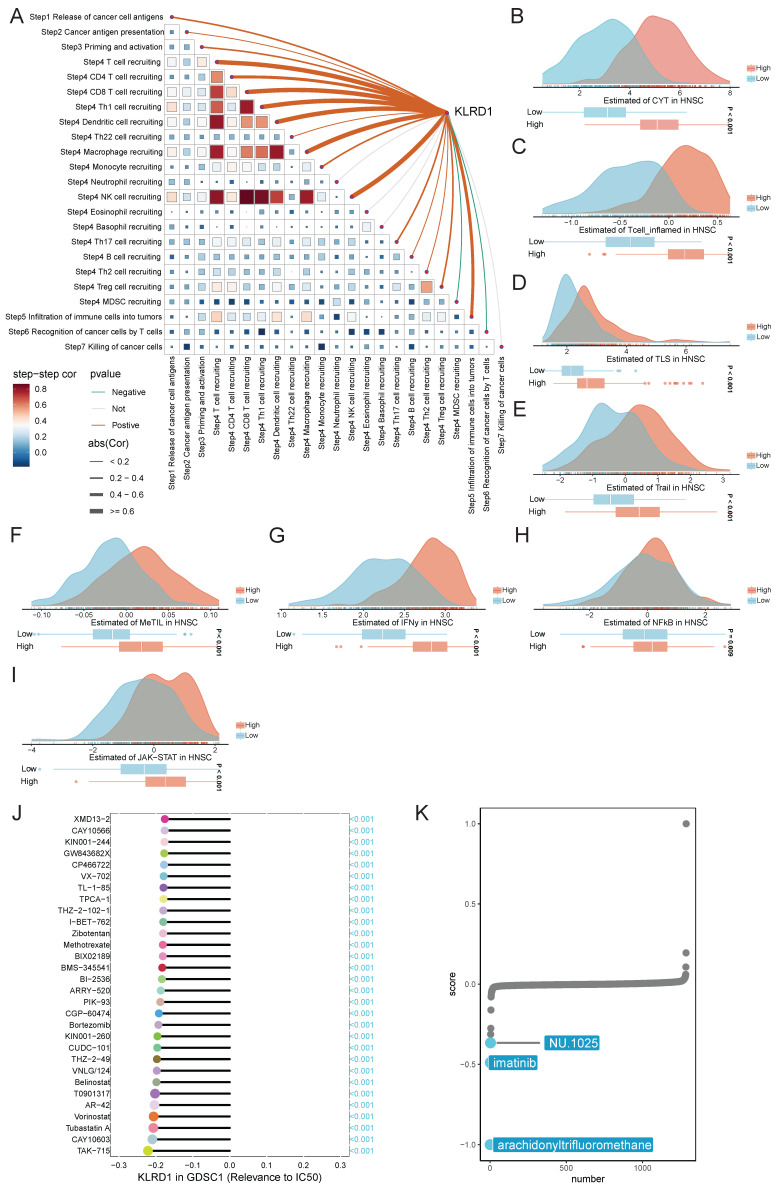
Analysis of KLRD1 in Cancer Immunity Cycle and Drug Sensitivity in HNSC. (A) Correlation analysis between KLRD1 expression and various steps of the cancer immunity cycle in HNSC. (B-I) Predicted score distributions of CYT, T-cell inflammation, TLS, T-cell tail, MeTIL, IFN-γ, NF-κB, and JAK-STAT pathways in HNSC based on high and low KLRD1 expression groups. (J) Correlation analysis of KLRD1 expression with IC50 values of various drugs in the GDSC1 dataset. (K) Ranking of drugs significantly correlated with KLRD1 based on drug sensitivity data.

The anticancer immune response comprises seven sequential steps: release of cancer cell antigens, antigen presentation, immune cell priming and activation, immune cell trafficking to the tumor, infiltration, recognition by T cells, and cancer cell killing 20. TIP meta-server integrates "ssGSEA" and "CIBERSORT" to analyze and visualize the anticancer immune status across these steps using RNA-seq or microarray data. Spearman correlation analysis revealed that in HNSC, KLRD1 is positively correlated with immune cell trafficking (Step 4) but predominantly negatively correlated with other steps of the cancer immunity cycle (**Figure 7A**). Additionally, the Easier tool, based on a cancer-specific immune response model, predicts immunotherapy responses using RNA-seq data 21. High KLRD1 expression is linked to increased activity of immune microenvironment indicators, including cytotoxic T cells, inflammatory T cells, tumor-infiltrating lymphocytes, and tumor-associated lymphoid structures, further underscoring its potential in boosting immune responses.

Spearman correlation analysis of gene expression and drug sensitivity using GDSC databases revealed that higher KLRD1 expression is associated with increased sensitivity to cell division inhibitors, with TAK-715, CAY10603, and Tubastatin A showing the strongest negative correlations with the half-maximal inhibitory concentration (IC50). Additionally, connectivity map (cMAP) analysis identified arachidonyltrifluoromethane as a potential compound that may counteract the oncogenic effects of dysregulated KLRD1 expression (**Figure 7J-K**). In HNSC, KLRD1 expression is closely associated with immune response regulation and drug sensitivity, highlighting its potential as a key biomarker for forecasting the response to specific anticancer drugs.

## 4. Discussion

Cancer is a major disease that poses a serious threat to human health and significantly affects prognosis 1, 2, 22, 23. Despite progress in traditional therapies like surgery, radiotherapy, and chemotherapy, overall outcomes remain less than idea 24. In recent years, immunotherapy, particularly immune checkpoint blockade, has gained prominence due to its potential to improve patient survival rates substantially 25, 26. HNSC, the sixth most common malignancy worldwide, is characterized by high rates of local recurrence, lymph node metastasis, and treatment failure, resulting in poor outcomes in advanced stages 27. Immunotherapy has emerged as a promising treatment option for HNSC 28-30. However, only a small subset of patients benefits from this approach 31, underscoring the need for novel therapeutic biomarkers to better guide immunotherapy strategies in HNSC.

KLRD1 is critical in regulating immune functions and tumor immune evasion within the tumor microenvironment 9, 11, 12. Exploring the potential of KLRD1 as a novel immune checkpoint is of great clinical significance. We systematically analyzed the expression characteristics of KLRD1 across various cancer types and its associations with genomic instability, immune microenvironment, and clinical prognosis. The results indicate that KLRD1 exhibits significant differential expression in multiple cancer types, with particularly strong relevance in HNSC, where its high expression correlates with better prognosis. These findings provide robust support for KLRD1 as a potential biomarker and therapeutic target, highlighting its critical role in cancer development, progression, and immune regulation.

TME is shaped by three key immune functions: immune clearance, immune balance, and immune evasion 32, 33. These processes are supported by a complex array of immune cells, including both adaptive and innate immune system components. Within the adaptive immune system, T cells, particularly CD8+ cytotoxic T cells and CD4+ helper T cells, play central roles. CD8+ T cells are essential for recognizing and eliminating tumor cells via MHC I, while CD4+ T cells, specifically Th1 cells, activate CD8+ T cells and NK cells to enhance antitumor activity 34-36. Our single-cell level analysis supports the high expression of KLRD1 in NK cells and CD8Tex cells, with particularly prominent expression in NK cells. Elevated expression of KLRD1 may play an important role in balancing tumor immune surveillance and immune evasion. KLRD1 expression is closely related to the infiltration of various immune cells, especially in HNSC, where these associations are more pronounced. Moreover, our pathway and functional enrichment analyses demonstrate that high KLRD1 expression is significantly enriched in pathways related to the immune-related diseases. This further elucidates the multiple roles of KLRD1 in regulating immune responses within the tumor microenvironment. Notably, KLRD1 expression varies significantly among different immune subtypes of HNSC, with particularly high expression in subtypes associated with IFN-γ signaling and tissue repair.

Our study reveals that KLRD1 plays a multifaceted role in the anticancer immune response and drug sensitivity in HNSC. Analysis based on the cancer immunity cycle shows that KLRD1 is positively associated with immune cell trafficking but negatively correlated with other steps, such as immune cell priming, activation, and cancer cell killing. This suggests that while KLRD1 may facilitate the movement of immune cells towards tumors, its role in other immune processes could be more complex. Supporting this, high KLRD1 expression correlates with increased activity of key immune components, including cytotoxic T cells, tumor-infiltrating lymphocytes, and inflammatory T cells, potentially enhancing the efficacy of immunotherapy. Additionally, findings from drug sensitivity analyses indicate that higher KLRD1 expression is linked to greater sensitivity to cell division inhibitors, such as TAK-715, CAY10603, and Tubastatin A. The identification of arachidonyltrifluoromethane as a potential countermeasure for dysregulated KLRD1 expression further opens new therapeutic possibilities. Overall, KLRD1 involvement in immune regulation and drug response highlights its potential in targeted treatments in HNSC, though its complex role in immune processes warrants further investigation.

Through the development of monoclonal antibodies targeting KLRD1, the binding between KLRD1 and its ligands can be blocked, thereby enhancing the anti-tumor activity of NK cells and T cells. For example, NKG2A, a subtype of KLRD1, has shown potential efficacy in various tumor types when targeted with specific antibodies 19, 37, 38. Additionally, combining KLRD1-targeted therapies with other immunotherapies, such as immune checkpoint inhibitors, may significantly improve treatment outcomes. Studies have demonstrated that the combination of NKG2A monoclonal antibodies with PD-1 inhibitors effectively activates the immune system and enhances anti-tumor responses 39. Furthermore, vaccines targeting KLRD1 can activate specific immune responses, improving the body's immune surveillance against tumors In iNKT cell therapy, introducing single-chain antibodies targeting KLRD1 into T cells enables them to more effectively recognize and attack tumor cells expressing the relevant ligands 40, 41. Although KLRD1-targeted immunotherapy is still in a developmental stage, researchers are actively exploring its potential applications across different cancer types and addressing possible resistance issues. Advances in this area offer new hope for cancer treatment.

Despite the important biological functions and clinical implications of KLRD1 revealed in this study, several limitations remain. Firstly, although we conducted extensive data analysis using public databases, these findings need further validation in larger clinical cohorts. Additionally, future studies should aim to validate these findings through experimental approaches.

## Supplementary Material

Supplementary figure.
