# Supplementary material for: KLRD1 (CD94): A Prognostic Biomarker and Therapeutic Candidate in Head and Neck Squamous Cell Carcinoma
Source: J Cancer. 2025 Jan 1;16(3):982–95. doi: 10.7150/jca.104762 (PMC11705060; doi:10.7150/jca.104762)
Supplement: Supplementary file 1 — Supplementary figure. [file jcav16p0982s1.pdf]

Supplemental Figure 1:

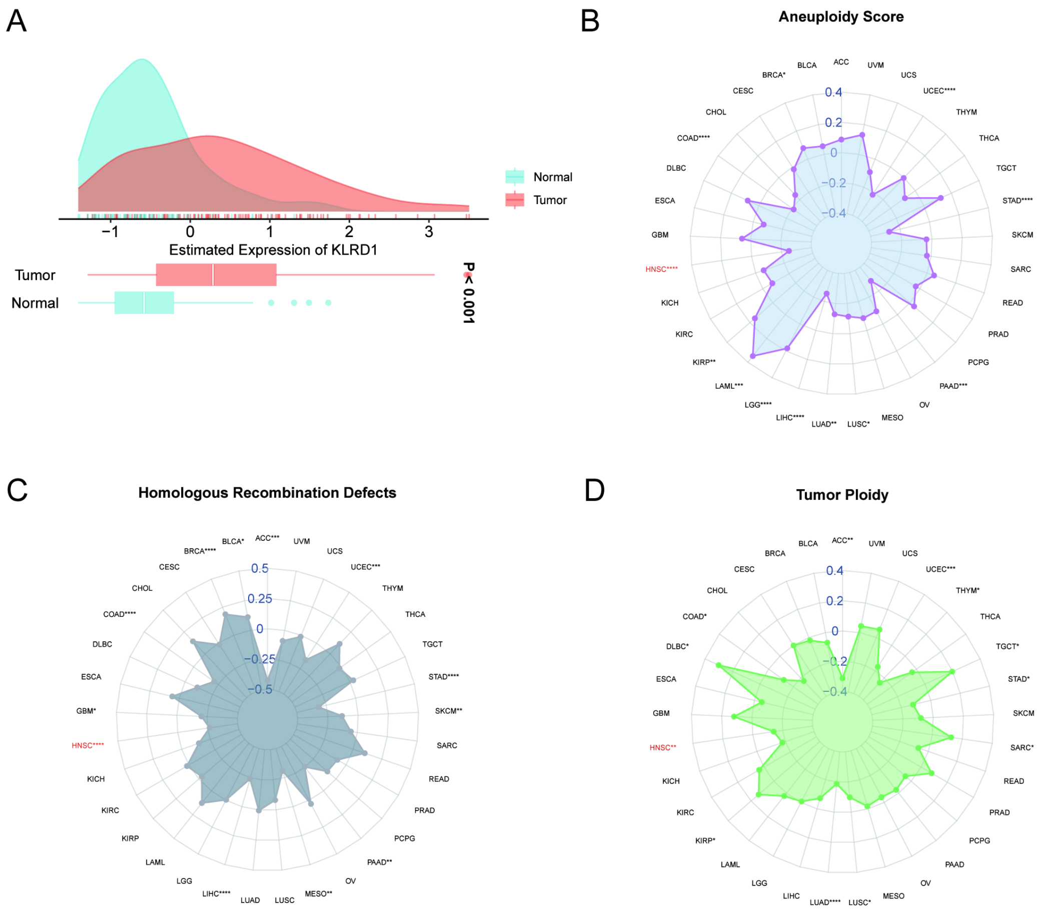

Supplemental Figure 1:

Analysis of KLRD1 Expression in HNSC and its Correlation with Genomic Stability. (A) Validation of KLRD1 expression in HNSC compared to normal tissues using the GEO dataset (E_MTAB_8588). (B) Correlation between KLRD1 expression and aneuploidy score in HNSC. (C) Correlation between KLRD1 expression and homologous recombination defects in HNSC. (D) Correlation between KLRD1 expression and ploidy score in HNSC.
